# The Effectiveness and Safety of Immune Checkpoint Inhibitors in Non-Small Cell Lung Cancer Patients With Stage III/IV: A Multicenter Study

**DOI:** 10.3389/fonc.2021.671127

**Published:** 2021-07-07

**Authors:** Jason C. Hsu, Phung-Anh Nguyen, Yen-Tzu Chen, Szu-Chun Yang, Chien-Chung Lin, Yi-Hsin Yang, Yu-Chao Lin, Te-Chun Hsia, Hsing-Chun Hsieh, Jia-Syuan Wu, Chi-Pei Chang, Yin-Hsun Feng, Peng-Chan Lin, Ping-Chih Hsu, Huey-En Tzeng, Shu-Chen Chien, Wei-Chiao Chang, Chih-Cheng Chang, Hsuan-Chia Yang, Chueh Ming Lee, Christine Y. Lu

**Affiliations:** ^1^ International Ph.D. Program in Biotech and Healthcare Management, College of Management, Taipei Medical University, Taipei, Taiwan; ^2^ International Center for Health Information Technology, College of Medical Science & Technology, Taipei Medical University, Taipei, Taiwan; ^3^ Department of Healthcare Information & Management, Ming Chuan University, Taoyuan, Taiwan; ^4^ School of Pharmacy and Institute of Clinical Pharmacy and Pharmaceutical Sciences, College of Medicine, National Cheng Kung University, Tainan, Taiwan; ^5^ Department of Internal Medicine, National Cheng Kung University Hospital, College of Medicine, National Cheng Kung University, Tainan, Taiwan; ^6^ National Institute of Cancer Research, National Health Research Institutes, Tainan, Taiwan; ^7^ Department of Respiratory Therapy, China Medical University Hospital, Taichung, Taiwan; ^8^ Department of Pharmacy, Chi-Mei Medical Center, Tainan, Taiwan; ^9^ Department of Internal Medicine, Chi-Mei Medical Center, Tainan, Taiwan; ^10^ Division of Hematology and Oncology, Department of Internal Medicine, Chi-Mei Medical Center, Tainan, Taiwan; ^11^ Division of Thoracic Medicine, Department of Internal Medicine, Linkou Chang Gung Memorial Hospital, Chang Gung University College of Medicine, Taoyuan, Taiwan; ^12^ Division of Hematology and Oncology, Department of Medicine, Taipei Medical University Hospital, Taipei, Taiwan; ^13^ Department of Clinical Pharmacy, College of Pharmacy, Taipei Medical University, Taipei, Taiwan; ^14^ School of Pharmacy, College of Pharmacy, Taipei Medical University, Taipei, Taiwan; ^15^ Department of Internal Medicine, School of Medicine, College of Medicine, Taipei Medical University, Taipei, Taiwan; ^16^ Graduate Institute of Biomedical Informatics, College of Medical Science & Technology, Taipei Medical University, Taipei, Taiwan; ^17^ Department of Family Medicine, National Cheng Kung University Hospital, Tainan, Taiwan; ^18^ Department of Population Medicine, Harvard Medical School and Harvard Pilgrim Health Care Institute, Boston, MA, United States

**Keywords:** effectiveness, immune checkpoint inhibitors (ICI), non-small-cell lung cancer (NSCLA), observational study, Taiwan

## Abstract

Immune checkpoint inhibitors (ICIs) have been approved to treat patients with various cancer types, including lung cancer, in many countries. This study aims to investigate the effectiveness and safety of ICIs under different treatment conditions of non-small cell lung cancer patients. A population-based retrospective cohort study was conducted using the electronic health records of three medical centers in Taiwan. From January 01, 2016, to November 30, 2018, a total of 91 ICIs and 300 traditional chemotherapy users who had undergone stage III and IV lung cancer treatment were included in the study. We performed the randomized matched pair design by selecting a Chemotherapy subject for each ICI patient in the sample population. All subjects were monitored from the date of taking ICIs or chemotherapy drugs until the event of death, loss to follow-up, or were occurred with any defined adverse events. Kaplan-Meier estimators and cox proportional hazard regression models were used to compute the overall survival, efficacy, and safety of the ICIs group. The median overall survival (OS) in the ICI and Chemo groups after matching was 11.2 months and 10.5 months, respectively. However, the results showed no significant OS differences between ICIs and chemo groups for both before and after matching (HR,1.30; 95%CI, 0.68-2.46; p=0.428 before matching and HR,0.96; 95CI%, 0.64-1.44; p=0.838 after matching). We observed that with the higher amount of PD-L1, the length of the patients’ overall survival was (positive *vs.* negative PD-L1, HR,0.21; 95%CI, 0.05-0.80; p=0.022). The incidences of serious adverse drug events above grade 3 in the ICIs and traditional chemo groups were 12.7% and 21.5%, respectively. We also found that the number of AEs was less in ICIs than in the Chemo group, and the AEs that occurred after treatments were observed earlier in the ICIs compared to the Chemo group. ICIs drugs were observed to be safer than traditional chemotherapy as they had a lower risk of serious adverse drug events. It is necessary to pay attention to immune-related side effects and provide appropriate treatment. Furthermore, the patient’s physical status and PD-L1 test can be used to evaluate the clinical effectiveness of ICIs.

## Introduction

Lung cancer is the most leading cause of cancer death worldwide, including in Taiwan (1, 2). Non-small-cell lung cancer (NSCLC) accounts for 85% of overall lung cancer patients (3, 4). In recent years, immune checkpoint inhibitors (ICIs) have been approved to treat patients with various cancer types in many countries (5, 6). Immunotherapy drugs work by blocking checkpoint proteins from binding with their associated proteins. Thus, it targets cancer cells to slow cells’ growth, prevent the cancer cell from spreading to other parts of the body, and increase the immune system’s effectiveness (6, 7).

Recently, clinical trials studies have reported that ICIs drugs (e.g., pembrolizumab, atezolizumab, and nivolumab) used independently or its combination with traditional chemotherapies in NSCLC patients were significantly associated with 2 to 4 months longer of overall survival (OS) or progression-free survival (PFS) compared to those used only platinum-based chemotherapy (8–10). Studies also showed that ICIs drug users significantly improved the objective response rate (ORR, 63.5% *vs.* 48%) (11); It, however, increased the number of adverse events (AEs) (e.g., AE of grade 3 and over, 55.7% *vs.* 47%) compared to chemotherapy users (9, 11) (see **Table S1** in the appendix). Besides, NSCLC patients without EGFR/ALK mutations or patients with PD-L1 expression over 5% of tumor cells were observed with a significantly higher ORR and less AEs among ICIs drug patients than chemotherapy alone (12).

Furthermore, clinical trials had their limitations that could not be seen in clinical practice (e.g., patients with comorbidities, be short-time follow-up, analyze the long-term AEs associated with drugs, and patients with different genotypes, ethnic groups), may lead to being differences in the long-term effect and safety of drugs, especially for the immunotherapeutic results (5, 8, 9). Bagley et al. (13) showed NSCLC patients with nivolumab had a 19.4% ORR, the OS of 6.5 months, and the PFS of 2.1 months in the USA. Manrique et al. (14) and Oya et al. (15) conducted the study to observe the OS, SFS of patients treated with nivolumab in Spain and Japan, respectively. Lin et al. (16) and Hsu et al. (17) reported the studies in Taiwan among the patients with either pembrolizumab or nivolumab had 32% ORR, the OS ranged from 7.9 to 13 months, and the PFS ranged from 1.8 to 4.9 months. However, those studies reported with lack of information, such as no control group (i.e., chemotherapy), did not include new therapy ‘atezolizumab’.

Therefore, this study aimed to address these gaps by examining the effectiveness and safety of ICIs under various treatment conditions and investigate the factors associated with their efficacy and safety. We also discuss the similarity and variations between our real-world empirical results and those obtained in previous clinical trials.

## Method

### Study Design and Data Source

We conducted a retrospective cohort study by retrieving all patients were undergone treatments of non-small cell lung cancer (NSCLC) with stage III and IV from three medical centers in Taiwan, including National Cheng Kung University Hospital, Chi Mei Hospital, and China Medical University Hospital. We obtained the data of patients from three electronic medical records between 2016 and 2018. This study has been approved by the institutional review boards (IRBs) of the National Cheng Kung University Hospital. The data was anonymized and de-identified before the analysis.

### Study Population

We identified patients diagnosed with lung cancer (International Classification of Disease, Tenth Revision, Clinical Modification [ICD-10-CM] codes C34), underwent treatments of NSCLC at stage III and IV from January 01, 2016, to November 30, 2018. We considered if patients had ever used immunological checkpoint inhibitors (ICIs) and those who had never used it in an analysis. Patients who had participated in any clinical trials and those who used more than two types of ICIs were excluded. Furthermore, patients with ages less than 20 years old were also excluded from the study.

Immunological checkpoint inhibitors (ICIs) drugs were classified as Anatomical Therapeutic Chemical (ATC) codes pembrolizumab (L01XC18), nivolumab (L01XC17), and atezolizumab (L01XC32) (see **Table S1** in the appendix). Cancer patients had received ICIs drugs for more than 14 days, defined as the ICIs group, compared with those who only used chemotherapy drugs.

To mimic the bias between two comparison groups, we performed the randomized matched-pair design. For each ICI patient, we selected a Chemo subject in the sample population; the randomized matched pair was matched for sex, age, EGFR/ALK mutation, daily performance status score (ECOG: 0-1 or 2-4 points), treatment situation (first-, second-, or third-line, or later).

### Main Outcome Measurements

All subjects were monitored from the date of taking ICIs or chemotherapy drugs. Data were censored at the date of death or the date of any adverse events (AEs) that occurred (e.g., Skin rash, fatigue/asthenia, colitis, diarrhea, hepatitis, constipation, pneumonitis, anorexia/decreased appetite, hypothyroidism, nausea, hyperthyroidism, vomiting, adrenal insufficiency, mucositis, and myositis) (**Table S5** in the appendix), loss to follow-up, termination of insurance, or the end of the study at December 31, 2018.

### Measurement of Covariates

We collected all information that might be associated with the mortality of studied patients. The data included demographic characteristics (e.g., age, sex, date of medical treatments, health performance status, smoking behavior status, HBsAg, and HCVAb), disease features (e.g., date of diagnosis, histology, stage, brain metastasis, EGFR/ALK mutation, and PD-L1 expression), and drug exposure information (e.g., line of treatments, drug types, a combination of chemotherapy, and baseline systemic steroid use). All covariates would include analyzing the effectiveness and safety of ICIs drugs in the study.

### Statistical Analysis

We used the modified Kaplan-Meier and Gray methods (18) to compare the cumulative probabilities in competing for both ICIs and Chemo groups’ overall survival in both samples, before and after matching. The log-rank test was used to estimate the differences in the time to event between patients using ICIs and Chemotherapy. In addition, hazard ratios (HRs) with 95% confidence intervals (CI) associated with the ICIs group were computed using Cox proportional hazard regression in competing for risk of death. Besides, we analyzed the factors related to the effectiveness and safety of ICI drugs.

All data management was performed using SAS v.9.3 software (SAS Institute Inc.). The statistical significance was considered with a p-value <0.05.

## Results

### Basic Characteristics of Study Patients

**Figure 1:**
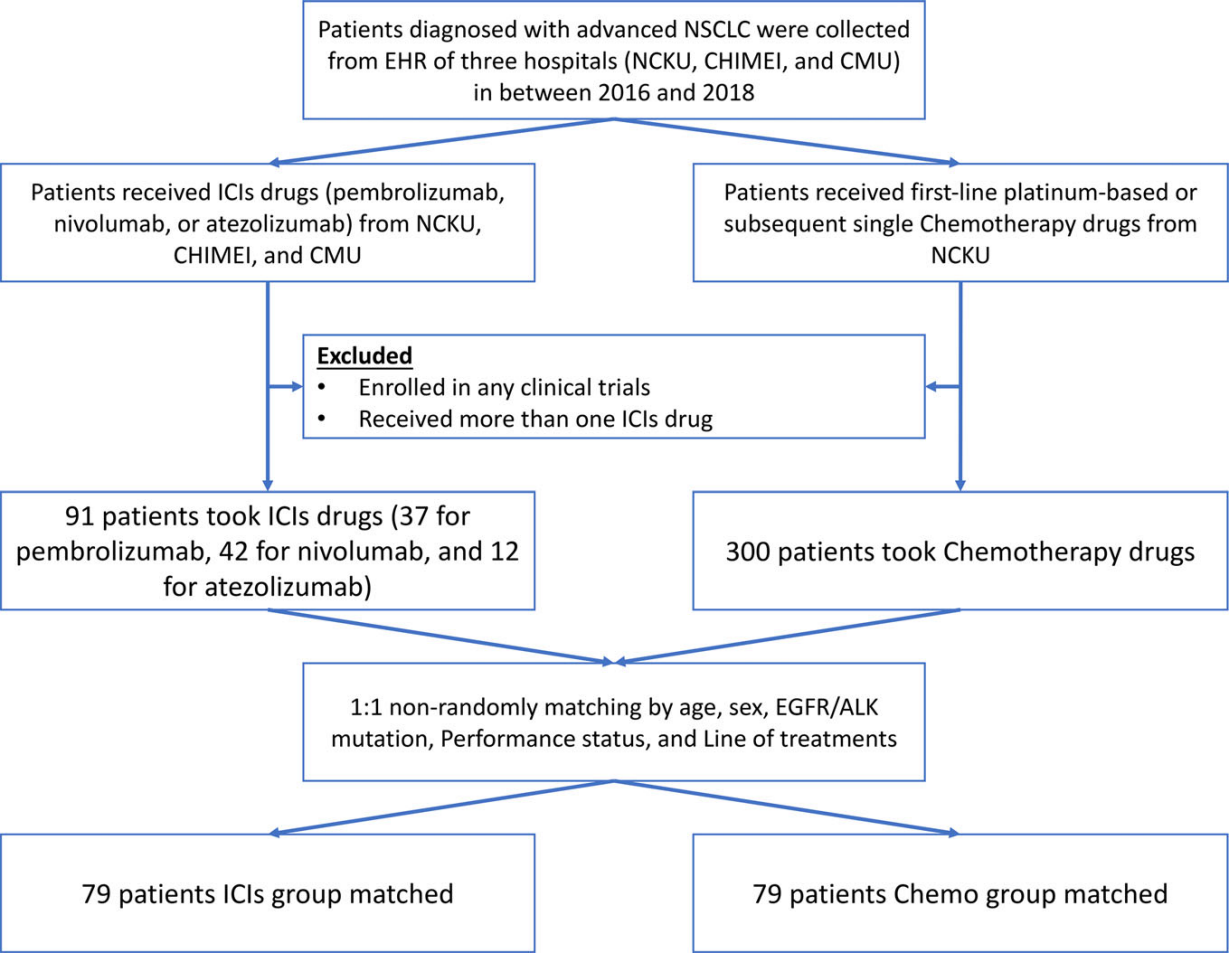
Enrollment process of the study population.

We identified 391 potentially eligible NSCLC patients from three hospital datasets between 2016 to 2018, in which 91 patients were taken ICIs, and 300 patients were treated with chemotherapy drugs. After matching, 158 patients were included in the further analysis, in which 79 patients were in the ICIs, and another 79 patients were in the Chemo group (see **Figure 1**).

Basic characteristics of study groups after matching were presented in **Table 1**. The mean (SD, standard deviation) age of patients in ICIs and Chemo groups were 63.9 (10.2) and 64.2 (10.2) years, respectively. Male NSCLC patients were observed more than female in both groups (i.e., 65.8% *vs.* 34.2%). In this study, the histological type of NSCLC patients was non-squamous cell carcinoma (i.e., 84.8% for ICIs and 81% for the Chemo group). For both groups, patients were at stage IV (91.1% for ICIs *vs.* 88.6% for Chemo), and most patients did not have brain metastases at the initial cancer diagnosis. In addition, the positive PD-L1 expression was observed higher in both ICIs and Chemo groups for those who performed the PD-L1 gene test. The detailed demographic characteristics of both groups by various treatment lines before matching has shown in **Table S3** in the appendix.

**Table 1:** Demographic characteristics of study population after matching.

Characteristics	Immune checkpoint inhibitors n = 79 (%)	Chemotherapy n = 79 (%)	p-value^a^
**Age**			0.370
Mean (SD)	63.9 (10.2)	64.2 (10.2)	
Age group			0.983
30-39	1 (1.3)	1 (1.3)	
40-49	5 (6.3)	5 (6.3)	
50-59	22 (27.8)	19 (24.1)	
60-69	28 (35.4)	32 (40.5)	
70-79	18 (22.8)	16 (20.3)	
80-89	5 (6.3)	6 (7.6)	
**Sex**			1.000
Male	52 (65.8)	52 (65.8)	
Female	27 (34.2)	27 (34.2)	
**Performance status**			1.000
0-1	69 (87.3)	69 (87.3)	
2-4	10 (12.7)	10 (12.7)	
**EGFR/ALK mutation**			1.000
Positive	20 (25.3)	20 (25.3)	
Negative	59 (74.7)	59 (74.7)	
**Treatment lines**			1.000
First-line	30 (42.9)	30 (42.9)	
Second-line	13 (16.5)	13 (16.5)	
Third-line and over	36 (45.6)	36 (45.6)	
**Histology**			0.526
Squamous	12 (15.2)	15 (19)	
Non-squamous	67 (84.8)	64 (81)	
Tumor stage^b^			0.598
III	7 (8.9)	9 (11.4)	
IV	72 (91.1)	70 (88.6)	
**Brain metastasis**			0.602
Yes	25 (31.6)	22 (27.8)	
No	54 (68.4)	57 (72.2)	
**PD-L1 expression**			<0.001
Positive	42 (53.16)	18 (22.8)	
Negative	7 (8.86)	6 (7.6)	
Missing	30 (38)	55 (69.6)	

a p-value was calculated using Student t test with continuous variables and chi-square or Fisher exact test with category variables.

b Tumor stage, represents the stage at the initial diagnosis of a cancer patient.

### Overall Survival Analysis of the Comparative Study Groups

**Figure 2:**
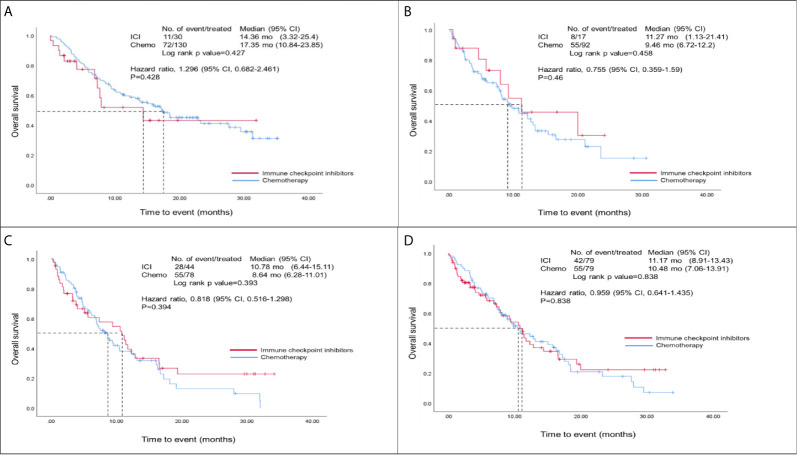
Overall survival analysis of ICIs and Chemo groups. **(A)** Overall survival analysis of the first-line group before matching; **(B)** Overall survival analysis of the second-line group before matching; **(C)** Overall survival analysis of those patients at third-line and over before matching; **(D)** Overall survival analysis of both ICIs and Chemo groups after matching; Hazard ratios were adjusted for histological types, tumor stage, brain metastasis, and PD-L1 expression variables in the cox-regression model.

**Figure 2** showed the overall survival analysis between ICIs and Chemo group. The overall survival was observed no significantly in NSCLC patients with ICIs drugs compared to those with Chemotherapy drugs (HR, 0.96; 95% CI, 0.64 – 1.44; p=0.838) after matching (**Figure 2D**). Furthermore, we found that there were no statistically significant differences for overall survival between ICIs and Chemo groups by various treatment lines (first-, second-, third-line and over) before/after matching analysis (i.e., in the first-line, HR, 1.30; 95% CI, 0.68 – 2.46; p=0.428 before matching and HR, 1.11; 95% CI, 0.51-2.43; p=0. 796 after matching) (**Figures 2A–C**, and **Table S4** in the appendix).

### Multivariable Stratified Analysis for the Efficiency and Safety of ICIs Drugs

**Table 2** showed the risk of death for the ICIs group after matching stratified by different factors. Patients with better performance status scores had a slightly reduced risk of death (i.e. 0-1 *vs.* 2-4, HR, 0.21; 95% CI, 0.05-1.01; p=0.051), while patients used steroid drugs had an increased risk of death (i.e. used *vs.* non-use, HR, 2.88; 95% CI, 0.92-9.07; p=0.071). We found that patients with positive PD-L1 expression gene tests had a significantly decreased risk of death than those with negative test results (HR, 0.21; 95% CI, 0.05-0.80; p=0.022). The detailed information of patients with ICIs drugs had shown in **Table S2** in the appendix.

**Table 2:** ICIs drugs use and its association with overall mortality by different covariates^a^.

Variables	Univariate		Multivariate	
	HR (95% CI)	p-value	HR (95% CI)^b^	p-value
**Sex**				
Male *vs.* Female	1.04 (0.58-1.87)	0.890	1.39 (0.48-4.05)	0.542
**Age**				
≥65 *vs.* <65	1.12 (0.63-1.99)	0.706	0.48 (0.16-1.51)	0.210
**Smoking status**				
Never *vs.* Current	2.41 (0.86-6.74)	0.093	1.86 (0.41-8.34)	0.419
**Performance status**				
0-1 *vs.* 2-4	0.22 (0.1-0.51)	<0.001	0.21 (0.05-1.01)	0.051
**Histology**				
Squamous *vs.* Non-squamous	0.90 (0.38-2.13)	0.813	0.78 (0.15-4.05)	0.765
**Brain metastasis**				
Yes *vs.* No	1.70 (0.94-3.08)	0.079	0.94 (0.32-2.73)	0.905
**PD-L1 expression**				
Positive *vs.* Negative	0.46 (0.18-1.19)	0.111	**0.21 (0.05-0.80)**	**0.022**
**EGFR/ALK mutation**				
Positive *vs.* Negative	1.26 (0.68-2.33)	0.463	0.74 (0.21-2.64)	0.638
**Treatment lines**				
First-line *vs.* Subsequent-line	0.78 (0.39-1.54)	0.468	1.26 (0.26-6.03)	0.770
**Steroid use**				
Yes *vs.* No	2.97 (1.59-5.57)	0.001	2.88 (0.92-9.07)	0.071
**Combined with chemotherapy**				
Yes *vs.* No	0.60 (0.32-1.10)	0.100	0.38 (0.11-1.30)	0.123
**Immune checkpoint inhibitors types**				
Pembrolizumab *vs.* Nivolumab	0.90 (0.46-1.75)	0.748	–	
Pembrolizumab *vs.* Atezolizumab	0.63 (0.27-1.51)	0.304	–	
Nivolumab *vs.* Atezolizumab	0.58 (0.26-1.33)	0.198	–	

HR, hazard ratio; CI, confidence intervals.

a Multivariable analysis is by Cox proportional hazards model.

b Adjusted for covariate factors, including in **Table S2** in the appendix. The bold values mean that it is a significant difference in the statistical analysis.

**Figure 3:**
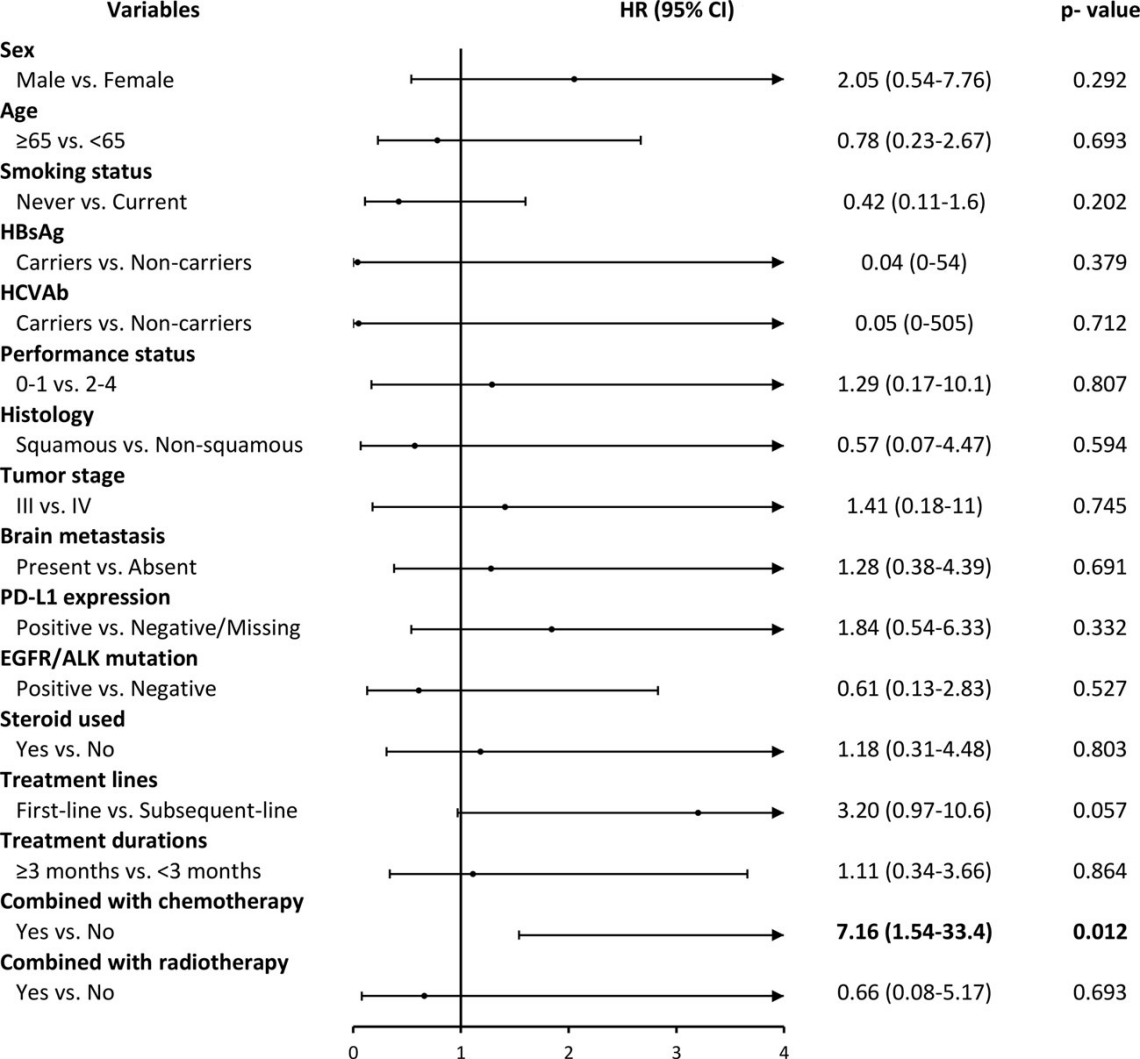
ICIs drugs use and its association with grade 3 and over adverse events by different covariates. HR, hazard ratio; CI, confidence intervals.

**Figure 3** showed the adverse events (AEs) for the ICIs group stratified by different factors. We observed that patients who used ICIs combined with chemotherapy drugs had significantly higher adverse events than those who did not use combination drugs (HR, 7.16; 95% CI, 1.54-33.4; p=0.012). However, we also found that the number of AEs was less in ICIs than in the Chemo group, and the AEs that occurred after treatments were observed earlier in ICIs compared to the Chemo group (7 *vs.* 13 months) (**Table S5** and **Figure S1** in the appendix).

## Discussion

This study is the first observational study in Taiwan to compare the real-world effectiveness and safety of ICIs and traditional chemotherapy in the treatment of NSCLC. It considered NSCLC patients who used ICIs at three medical centers in Taiwan. The median overall survival in the first-line treatment setting was 14.4 months in the ICIs group and 17.4 months in the Chemo group. In the study, the median overall survival of the ICIs group was similar to the clinical trial results; the survival rate, however, was significantly longer in the Chemo group compared to those were in the clinical trial studies (17.4 *vs.* 11.3-14.7 months). The mortality risk ratio was 1.3 (95% CI, 0.68-2.46), with no significant between the comparative groups. The study subjects were Asian, with the proportion in the clinical trials was relatively low (1-19.4%) (19). Furthermore, studies have reported that Asian NSCLC patients have better survival prognoses and treatment responses than their non-Asian counterparts (20–22). A separate analysis of first-line chemotherapy among patients with NSCLC in Taiwan found that the median overall survival after treatment with different platinum-containing combined chemotherapy treatments ranged from 16.6 to 27.1 months (23), which is comparable to the results of the Chemo group in this study, suggesting that patients with NSCLC in Taiwan have reasonable survival rates with chemotherapy.

Furthermore, high PD-L1 expression (≥50%) is often used alone with pembrolizumab (9, 11), while low PD-L1 expression (<50%) has a better survival benefit when combined with pembrolizumab or atezolizumab and platinum-containing chemotherapy (24, 25). However, most of the drugs in the ICI group in this study were started before the relevant clinical trials were published; the treatment model was not consistent with the clinical trials, and the proportion of high PD-L1 expression (≥50%) was not as high as those in clinical trials.

Besides, we observed that it might take about three months or more from the start of ICIs to the response (10, 26–28), and the response pattern is different from that with chemotherapy. The immune system’s particular mechanism induced by ICI causes the treatment response to persist until the drug is discontinued. It may even cause temporary deterioration of the tumor due to the temporary infiltration of immune cells. The tumor may begin to shrink after the disease has been observed to worsen (29, 30). However, the ICI group’s median observation period in this study was only 3.94 months, which may not have been long enough to track the occurrence of treatment response.

### Survival Trend in Different Treatment Lines

In the second-line and third-line treatment settings, the median overall survival in the ICI group was about two months longer than that in the Chemo group (second-line: 11.3 *vs.* 9.5 months, HR: 0.76, P = 0.46; third-line or later: 10.8 *vs.* 8.6 months, HR: 0.84, P = 0.39). Although there were no statistically significant differences, a better survival trend was observed in the ICI group. The second-and third-line subjects in this study were more likely to carry EGFR or ALK mutations, especially in third-line or later treatments. Studies have suggested that the use of second-line ICIs alone in treating lung cancer patients with EGFR mutations does not lead to good survival benefits (31, 32). Good outcomes might be achieved when combined ICIs with chemotherapy. Chemotherapy is generally considered to have only immunosuppressive effects, such as bone marrow suppression or hemocytopenia. However, recent studies have found that chemotherapy may have immunomodulatory properties, which can be induced by the tumor microenvironment from the immune desert or immune excluded to a state conducive to the role of immune cells (33). Even if the proportion of patients with EGFR or ALK mutations increases, chemotherapy may provide some survival benefits (34).

### The Effectiveness and Safety of ICIs Drugs

Past clinical trials and observational studies have mentioned better physical performance status and high PD-L1 expression as factors that reduce death risk (16, 35, 36). In this study, PD-L1 expression was analyzed using stratification; Although there were no statistically significant differences, it was observed that the higher amount of PD-L1, the longer of the patient’s overall survival was. In a past Taiwan cohort study (16), 74 patients with advanced NSCLC treated with pembrolizumab or nivolumab alone were analyzed by stratifying PD-L1. The median overall survival also showed the same increasing trend found in this study (PD-L1 ≥50%, not reached; PD-L11-49%, 10.5 months; PD-L1 <1%, 13.2 months; p = 0.217). The results confirmed that PD-L1 expression could be used as an evaluation index for the clinical selection of drugs. It is worth noting that this study included more patients with poor physical performance (PS≥2, 36/74, 48.6%), where the ICI was third-line or later, accounting for 70%. The median overall survival of all patients was 7.9 months, which was worse than the overall survival of all patients in this study’s ICI group (11.2 months).

Regarding the effects of steroids on ICIs, a real-world study of patients with NSCLC in the USA used pembrolizumab, nivolumab, atezolizumab, or durvalumab alone at two cancer centers compared the effectiveness of oral or injectable steroids in the first 30 days of use of ICIs. Steroids reduce not only overall patient survival but also treatment response and non-deteriorating survival (37).

Hepatitis patients are usually excluded from clinical ICI trials. However, Taiwan is a region with a high prevalence of hepatitis B. There is insufficient data on the effectiveness of ICI in patients with cancer and hepatitis B. According to the stratified survival analysis results in this study, the overall survival of patients with hepatitis B was significantly lower. Chronic infection may inhibit the role of T cells, thereby reducing the effect of drug-induced immunity (38). Thus, clinicians should carefully evaluate the suitability of immunotherapy for cancer patients with comorbid hepatitis.

Safety results reported in the clinical trials have shown that ICIs cause fewer serious adverse drug events than chemotherapy. This study confirmed this finding, but the between-group differences were not significant (ICIs: 10 patients, 12.7% *vs.* Chemo: 17 patients, 21.5%). The type of adverse events in the ICI group were mainly immune-related side effects. The common types were pneumonitis and skin rash, similar to past clinical trials and observational studies (39, 40). In addition, this study found that adverse events in the two groups occurred early in the treatment. Even the Chemo group had the most occurrences within one month of starting the treatment, with no significant between-group differences. Past literature indicates that most immune-related side effects occur within six months after medication (41). The adverse events observed in the ICI group in this study all happened within seven months after initiation of treatment and showed similar results. Besides, in terms of the type of immune-related side effects, in the past, the skin and gastrointestinal tract were found to be the earliest affected organs, followed by the liver, lungs, and endocrine system (41, 42). However, due to the small number of incidents and the short observation period, the same performance trend was not observed in this study. Therefore, we need to include more patients and extend the observation period to confirm the adverse event performance of ICIs.

There have been no published studies comparing the safety of ICI alone with concurrent chemotherapy in NSCLC. However, one network integration analysis included four Phase III clinical trials of the first-line pembrolizumab, which indirectly compared the adverse event risk of pembrolizumab with grade 3-5 combined with chemotherapy alone. The results also showed that the risk of concomitant use significantly increased the risk of adverse events compared to a single-use (RR 2.14, 95% CI 1.50-3.05; P <0.001) (43).

### Limitations

This study has the following limitations. First, the number of patients in the ICI group was less may affect the outcomes. The ICI was licensed as early as December 2015, Taiwan Health Insurance did not cover it until April 2019. All patients have to pay for ICIs at their own expense before payment is approved. These drugs are expensive and require continuous treatment until the disease worsens or severe intolerable side effects occur (44). Even if the patient is expected to experience an excellent therapeutic effect (such as high PD-L1 expression), the enormous economic burden makes the number of patients willing to accept treatment with ICIs low.

Second, the data source of this study was the electronic medical records from three medical centers. Some items had to be evaluated by a clinician, such as physical performance scores, adverse drug events, severity, etc. Data collection may have been incomplete because the patient did not report ultimately, or the medical records were not noted with this data. In addition, the PD-L1 detection rate of this research object was low, and more test data is needed further to verify the impact of performance on overall survival.

Third, this study’s observation period was relatively short, especially in the ICI group, because nearly 41% of the patients started taking medication in 2018, and the median observation period was less than one year. Since most of the ICI clinical trials for NSCLC were published from 2016 to 2018 (10, 11, 45), the patients’ treatment model in the ICI group did not fully follow the clinical trials’ conclusion not show the actual effectiveness of the drug.

## Conclusion

The overall survival of patients with advanced NSCLC in Taiwan using ICIs was not significantly better than that for patients undergoing traditional chemotherapy, regardless of where it was a first-line or subsequent treatment. It was also observed that ICIs drugs have a lower risk of serious adverse drug events than traditional chemotherapy, which indicated that they are safer. Nevertheless, ICI combined with chemotherapy may increase the risk of occurrence of adverse events. Therefore, it is still necessary to pay attention to immune-related side effects and provide appropriate treatment as soon as possible. Furthermore, the patient’s physical status and PD-L1 test can be used to evaluate the clinical effectiveness of ICIs. Patients with hepatitis B receiving ICI and using systemic steroids at the beginning of treatment need to be carefully monitored for possible adverse reactions.

## Data Availability Statement

The raw data supporting the conclusions of this article will be made available by the authors, without undue reservation.

## Ethics Statement 

The studies involving human participants were reviewed and approved by National Cheng Kung University Hospital. Written informed consent for participation was not required for this study in accordance with the national legislation and the institutional requirements.

## Author Contributions 

P-AN, JCH, Y-TC, S-CY, C-CL, and Y-HY conceptualized and designed the study. P-AN, Y-CL, T-CH, Y-HF, P-CL, P-CH, H-ET, S-CC, and W-CC provided suggestions for the research design from a clinical perspective. Y-TC, Y-CL, H-CH, J-SW, and CML collected data, performed the analyses, and drafted the manuscript. P-AN and CYL reviewed all data and revised the manuscript critically for intellectual content. All authors contributed to the article and approved the submitted version.

## Funding

This work was supported by the Taiwan Ministry of Science and Technology grants [grant numbers: MOST 107-2320-B-006-040 and MOST 108-2314-B-006-039].

## Conflict of Interest

The authors declare that the research was conducted in the absence of any commercial or financial relationships that could be construed as a potential conflict of interest.

## Abbreviation

NSCLC, Non-small-cell lung cancer; ICIs, Immune checkpoint inhibitors; OS, Overall survival; PFS, Progression-free survival; ORR, Objective response rate; AEs, Adverse events; ICD-10-CM, International Classification of Disease, Tenth Revision, Clinical Modification; ATC, Anatomical Therapeutic Chemical; HRs, Hazard ratios; Cis, Confidence intervals; IRBs, Institutional review boards.
