# Supplementary material for: The Effectiveness and Safety of Immune Checkpoint Inhibitors in Non-Small Cell Lung Cancer Patients With Stage III/IV: A Multicenter Study
Source: Front Oncol. 2021 Jul 7;11:671127. doi: 10.3389/fonc.2021.671127 (PMC8293991; doi:10.3389/fonc.2021.671127)
Supplement: Supplementary file 1 [file DataSheet_1.docx]

**The Effectiveness and Safety of Immune Checkpoint Inhibitors in Non-Small Cell Lung Cancer Patients with Stage III/IV**

Supplementary Appendix

Table of Contents

[Table S1. Studies related to efficacy and safety of cancer immunotherapies (Clinical Trials) 2](#_Toc64971203)

[Table S2. Demographic characteristic of ICIs patients before matching 3](#_Toc64971204)

[Table S3. Basic characteristics of both ICIs and Chemo groups by treatment lines before matching 4](#_Toc64971205)

[Table S4. Overall survival analysis between ICIs and Chemo group by treatment lines after matching 5](#_Toc64971206)

[Table S5. Treatment-related adverse events in both ICIs and Chemo groups 6](#_Toc64971207)

[Figure S1. Treatment-related adverse events by time 7](#_Toc64971208)

# **Table S1**. Studies related to efficacy and safety of cancer immunotherapies (Clinical Trials)

| **Name of Trial** | **Drug name** | **Author** | **PD L1, %** | **With chemo** | **Line** | **N_E** | **N_C** | **Study Time** | **RR, %** | **Median OS, (month)** | **Median PFS, (month)** | **≥Grade 3 AE, %** |
| --- | --- | --- | --- | --- | --- | --- | --- | --- | --- | --- | --- | --- |
| KEYNOTE-021 | Pembrolizumab | Langer (2016) | All | V | 1st | 60 | 63 | 2014.11-2016.01 | 55 vs. 29 | - | 13 vs. 8.9 | 39 vs. 26 |
| IMpower150 | Atezolizumab | Socinski (2018) | - | V | 1^st^ | 400 | 400 | 2015.03-2016.12 | 63.5 vs. 48 | 19.2 vs. 17.4 | 8.3 vs. 6.8 | 55.7 vs. 47.7 |
| KEYNOTE-407 | Pembrolizumab | Paz-Ares (2018) | All | V | 1^st^ | 278 | 281 | 2016.08-2017.12 | 57.9 vs. 38.4 | 15.9 vs. 11.3 | 6.4 vs. 4.8 | 69.2 vs. 68.2 |
| KEYNOTE-189 | Pembrolizumab | Gandhi (2018) | All | V | 1^st^ | 410 | 206 | 2016.02-2017.03 | 47.6 vs. 18.9 | NR vs. 11.3 | 8.8 vs. 4.9 | 67.2 vs. 65.8 |
| KEYNOTE-024 | Pembrolizumab | Reck (2016) | ≥50% | X | 1^st^ | 154 | 151 | 2014.09-2015.10 | 44.8 vs. 27.8 | - | 10.3 vs. 6 | 26.6 vs. 53.3 |
| KEYNOTE-042 | Pembrolizumab | Mok (2019) | ≥50%, ≥20%, ≥1% | V | 1^st^ | 637 | 637 | 2014.12-2017.03 | - | 20.0 vs. 12.2;  17.7 vs. 13.0  16.7 vs. 12.1 | 7.1 vs. 6.4  6.2 vs. 6.6  5.4 vs. 6.5 | 17.8 vs. 41.0 |
| CheckMate-026 | Nivolumab | Carbone (2017) | ≥1% | X | 1st | 271 | 270 | 2014.03-2015.04 | 26 vs. 33 | 14.4 vs. 13.2 | 4.2 vs. 5.9 | 18 vs. 51 |
| OAK | Atezolizumab | Rittmeyer (2017) | All | X | 2^nd^ | 425 | 425 | 2014.03-2015.04 | 14 vs. 13 | 13.8 vs. 9.6 | 2.8 vs. 4 | 15 vs. 43 |
| CheckMate-017 | Nivolumab | Brahmer (2015) | All | X | 2^nd^ | 135 | 137 | 2012.10-2013.12 | 20 vs. 9 | 9.2 vs. 6 | 3.5 vs. 2.8 | 7 vs. 55 |
| CheckMate -057 | Nivolumab | Borghaei (2015) | All | X | 2^nd^ | 292 | 290 | 2012.11-2013.12 | 19 vs. 12 | 12.2 vs. 9.4 | 2.3 vs. 4.2 | 10 vs. 54 |
| KEYNOTE-010 | Pembrolizumab | Herbst (2016) | >1% | X | 2^nd^ | 344(2mg/kg); 346(10mg/kg) | 343 | 2013.08-2015.02 | - | 10.42(2mg/kg); 12.7(10mg/kg) vs. 8.5 | 3.9(2mg/kg); 4(10mg/kg) vs. 4 | 13(2mg/kg); 16(10mg/kg) vs. 35 |

**Note**: N_E, number of experimental group; N_C, number of control group; RR, response rate; OS, overall survival; PFS, progress-free survival; AE, adverse events;

# **Table S2.** Demographic characteristic of ICIs patients before matching

| **Variables** | **Immune checkpoint inhibitors,  n = 91, N (%)** | **Variables** | **Immune checkpoint inhibitors,  n = 91, N (%)** |
| --- | --- | --- | --- |
| **Age** |  | **Smoking status** |  |
| Mean (SD) | 62.87 (11.54) | Current | 17 (18.7) |
| 30-39 | 2 (2.2) | Never | 74 (81.3) |
| 40-49 | 11 (12.1) | **HBsAg** |  |
| 50-59 | 22 (24.2) | Positive | 12 (13.2) |
| 60-69 | 29 (31.9) | Negative | 74 (81.3) |
| 70-79 | 21 (23.1) | Missing | 5 (5.5) |
| 80-89 | 6 (6.6) | **HCVAb** |  |
| **Sex** |  | Positive | 2 (2.2) |
| Male | 58 (63.7) | Negative | 82 (90.1) |
| Female | 33 (36.3) | Missing | 7 (7.7) |
| **Performance status** |  | **Lung cancer surgery history** |  |
| 0-1 | 79 (86.8) | Yes | 16 (17.6) |
| 2-4 | 12 (13.2) | No | 75 (82.4) |
| **EGFR/ALK mutation** |  | **Steroid use^c^** |  |
| Positive | 22 (24.2) | Yes | 19 (20.9) |
| Negative | 68 (74.7) | No | 72 (79.1) |
| **Treatment lines^a^** |  | **ICIs types** |  |
| First-line | 30 (33) | Pembrolizumab | 37 (40.7) |
| Second-line | 17 (18.7) | Nivolumab | 42 (46.2) |
| Third-line and over | 44 (48.4) | Atezolizumab | 12 (13.2) |
| **Histology** |  | **Median treatment duration, month** |  |
| Squamous | 15 (16.5) | Pembrolizumab | 2.07 |
| Non-squamous | 76 (83.5) | Nivolumab | 3.44 |
| **Tumor stage^b^** |  | Atezolizumab | 3.45 |
| III | 7 (7.7) | **Combined with chemotherapy** |  |
| IV | 84 (92.3) | Yes | 41 (45.1) |
| **Brain metastasis** |  | No | 50 (54.9) |
| Yes | 27 (29.7) | **Combined with radiotherapy** |  |
| No | 64 (70.3) | Yes | 10 (11) |
| **PD-L1 expression** |  | No | 81 (89) |
| Positive | 46 (50.5) | **Grade 3 and over of AE** |  |
| Negative | 8 (8.8) | Yes | 11 (12.1) |
| Missing | 37 (40.7) | No | 80 (87.9) |

**Note**: ^a^Treatment lines, represents the line of treatment with medications.

^b^Tumor stage, represents the stage of patients at the time of cancer diagnosis.

^c^Steroid used, represents the patients who used steroid drugs more than a week (7 days) at the time of cancer treatment.

# **Table S3.** Basic characteristics of both ICIs and Chemo groups by treatment lines before matching

| **Variables** | **First-line^a^** | | | **Second-line^a^** | | | **Third-line and over^a^** | | |
| --- | --- | --- | --- | --- | --- | --- | --- | --- | --- |
|  | **Immune checkpoint inhibitors**  **n = 30, N (%)** | **Chemotherapy**  **n = 130, N (%)** | **p-value^b^** | **Immune checkpoint inhibitors**  **n = 17, N (%)** | **Chemotherapy**  **n = 92, N (%)** | **p-value^b^** | **Immune checkpoint inhibitors**  **n = 44, N (%)** | **Chemotherapy**  **n = 78, N (%)** | **p-value^b^** |
| **Age** |  |  | 0.155 |  |  | 0.127 |  |  | 0.339 |
| Mean (SD) | 66.6 (12.2) | 62.02 (11.1) | **0.047** | 62 (9.5) | 67.4 (11.1) | 0.063 | 60.05 (11.3) | 63.79 (11.0) | 0.075 |
| 30-39 | 1 (3.3) | 5 (3.8) |  | 0 (0) | 1 (1.1) |  | 2 (4.5) | 1 (1.3) |  |
| 40-49 | 1 (3.3) | 10 (7.7) |  | 2 (11.8) | 5 (5.4) |  | 7 (15.9) | 5 (6.4) |  |
| 50-59 | 8 (26.7) | 33 (25.4) |  | 3 (17.6) | 17 (15.8) |  | 12 (27.3) | 25 (32.1) |  |
| 60-69 | 6 (20) | 51 (39.2) |  | 10 (58.8) | 28 (30.4) |  | 12 (27.3) | 20 (25.6) |  |
| 70-79 | 10 (33.3) | 24 (18.5) |  | 2 (11.8) | 29 (31.5) |  | 10 (22.7) | 21 (26.9) |  |
| 80-89 | 4 (13.3) | 7 (5.4) |  | 0 (0) | 12 (13) |  | 1 (2.3) | 6 (7.7) |  |
| **Sex** |  |  | 0.815 |  |  | 0.552 |  |  | 0.751 |
| Male | 23 (76.7) | 97 (74.6) |  | 10 (58.8) | 61 (66.3) |  | 25 (56.8) | 42 (53.8) |  |
| Female | 7 (23.3) | 33 (25.4) |  | 7 (41.2) | 31 (33.7) |  | 19 (43.2) | 36 (46.2) |  |
| **Histology** |  |  | 0.517 |  |  | 0.897 |  |  | 0.983 |
| Squamous | 7 (23.3) | 38 (29.2) |  | 4 (23.5) | 23 (25) |  | 4 (9.1) | 7 (9) |  |
| Non-squamous | 23 (76.7) | 92 (70.8) |  | 13 (76.5) | 69 (75) |  | 40 (90.9) | 71 (91) |  |
| **Stage** |  |  | **0.004** |  |  | 0.374 |  |  | 0.185 |
| III | 1 (3.3) | 37 (28.5) |  | 2 (11.8) | 20 (21.7) |  | 4 (9.1) | 14 (17.9) |  |
| IV | 29 (96.7) | 93 (71.5) |  | 15 (88.2) | 72 (78.3) |  | 40 (90.9) | 64 (82.1) |  |
| **Performance status** |  |  | 0.324 |  |  | 0.430 |  |  | 0.062 |
| 0-1 | 26 (86.7) | 120 (92.3) |  | 14 (82.4) | 63 (68.5) |  | 39 (88.6) | 55 (70.5) |  |
| 2-4 | 4 (13.3) | 10 (7.7) |  | 3 (17.7) | 23 (25) |  | 5 (11.4) | 19 (24.4) |  |
| **EGFR/ALK mutation** |  |  | 0.630 |  |  | 0.825 |  |  | 0.125 |
| Positive | 0 (0) | 1 (0.8) |  | 4 (23.5) | 24 (26.1) |  | 18 (40.9) | 44 (56.4) |  |
| Negative | 30 (100) | 129 (99.2) |  | 13 (76.5) | 68 (73.9) |  | 25 (56.8) | 34 (43.6) |  |
| **PD-L1 expression** |  |  | **<0.001** |  |  | **0.027** |  |  | **0.012** |
| Positive | 23 (76.7) | 35 (26.9) |  | 9 (52.9) | 20 (21.7) |  | 11 (25) | 16 (20.5) |  |
| Negative | 1 (3.3) | 10 (7.7) |  | 1 (5.9) | 7 (7.6) |  | 6 (13.6) | 1 (1.3) |  |
| Missing | 6 (20) | 85 (65.4) |  | 7 (41.2) | 65 (70.7) |  | 27 (61.4) | 61 (78.2) |  |

**Note**: ^a^Treatment lines, represents the initial line of treatment with medications.

^b^p-value was calculated using Student t test with continuous variables and chi-square or Fisher exact test with category variables.

# **Table S4.** Overall survival analysis between ICIs and Chemo group by treatment lines after matching

|  | **Unadjusted** | | **Adjusted** | |
| --- | --- | --- | --- | --- |
|  | **HR (95% CI)** | **p-value** | **HR (95% CI)** | **p-value** |
| All line | 0.96 (0.64-1.44) | 0.838 | 0.93 (0.64-1.44) | 0.838 |
| First-line | 1.1 (0.51-2.35) | 0.812 | 1.11 (0.51-2.43) | 0.796 |
| Second-line | 1.04 (0.35-3.12) | 0.947 | 0.95 (0.28-3.19) | 0.931 |
| Third-line and over | 0.75 (0.43-1.32) | 0.319 | 0.81 (0.45-1.45) | 0.477 |

**Note**: The adjusted hazard ratio was adjusted for histological types, Tumor stage, Brain metastasis, and PD-L1 expression variables

# **Table S5.** Treatment-related adverse events in both ICIs and Chemo groups

| **Adverse event types** | **Immune checkpoint inhibitors**  **n = 79, N (%)** | **Chemotherapy**  **n = 79, N (%)** |
| --- | --- | --- |
| **Overall** | 10 (12.7) | 17 (21.5) |
| Pneumonitis | 5 (6.3) | 0 |
| Hepatitis | 1 (1.3) | 0 |
| Hyperthyroidism | 1 (1.3) | 0 |
| Multiple arthralgia | 1 (1.3) | 0 |
| Pruritus | 1 (1.3) | 0 |
| Encephalitis | 1 (1.3) | 0 |
| Chronic inflammatory | 1 (1.3) | 0 |
| Demyelinating polyneuropathy |  |  |
| Fatigue | 2 (2.5) | 2 (2.5) |
| Skin rash | 3 (3.8) | 0 |
| Neutropenia | 0 | 10 (12.7) |
| Alopecia | 0 | 3 (3.8) |
| Anemia | 0 | 2 (2.5) |
| Diarrhea | 0 | 1 (1.3) |
| Nausea | 0 | 1 (1.3) |
| Vomiting | 0 | 1 (1.3) |
| Numbness | 0 | 1 (1.3) |
| Septic cardiomyopathy | 0 | 1 (1.3) |
| Septic shock | 0 | 1 (1.3) |
| Anorexia | 0 | 1 (1.3) |

| A.  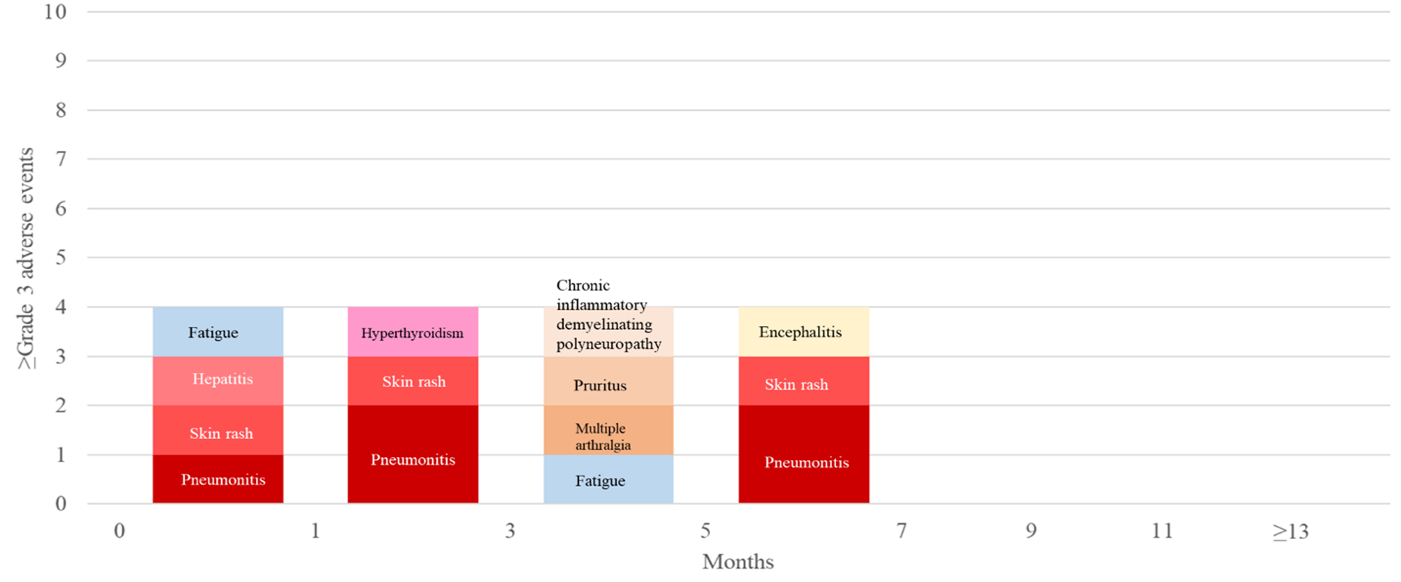 |
| --- |
| B.  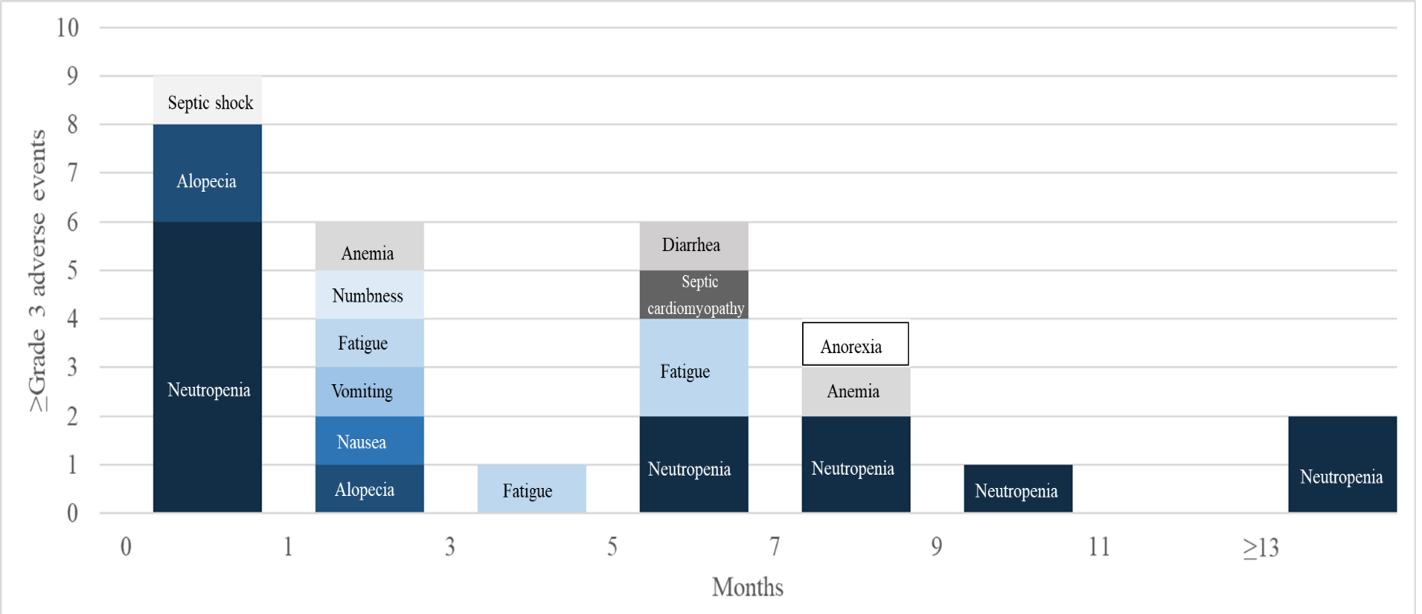 |

# **Figure S1.** Treatment-related adverse events by time

**Note**: A, ICIs drugs group; B, Chemotherapy drugs group
